# Supplementary material for: Genotype-Phenotype Correlation in Hypertrophic Cardiomyopathy: New Variant p.Arg652Lys in MYH7
Source: Genes (Basel). 2022 Feb 9;13(2):320. doi: 10.3390/genes13020320 (PMC8872101; doi:10.3390/genes13020320)
Supplement: Supplementary file 1 [file genes-13-00320-s001.zip › genes-1525944-supplementary.pdf]

Supplementary material

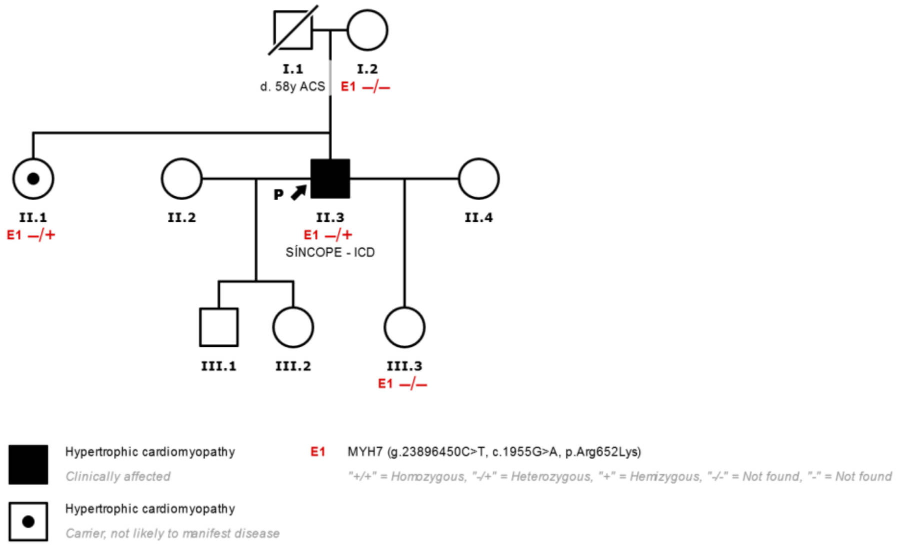

Figure 1. Family tree Nº 1. II.3 corresponds to index case Nº 1 in Table 1.
Full black patients correspond to HCM affected patients. Patients with the p.Arg652Lys in MYH7 variant and no HCM phenotype are marked with a central black mark. ACS: Acute coronary syndrome. ICD: Implantable Cardiac Defibrillator. - / -: Variant not found. - / +: Heterozygous variant found.

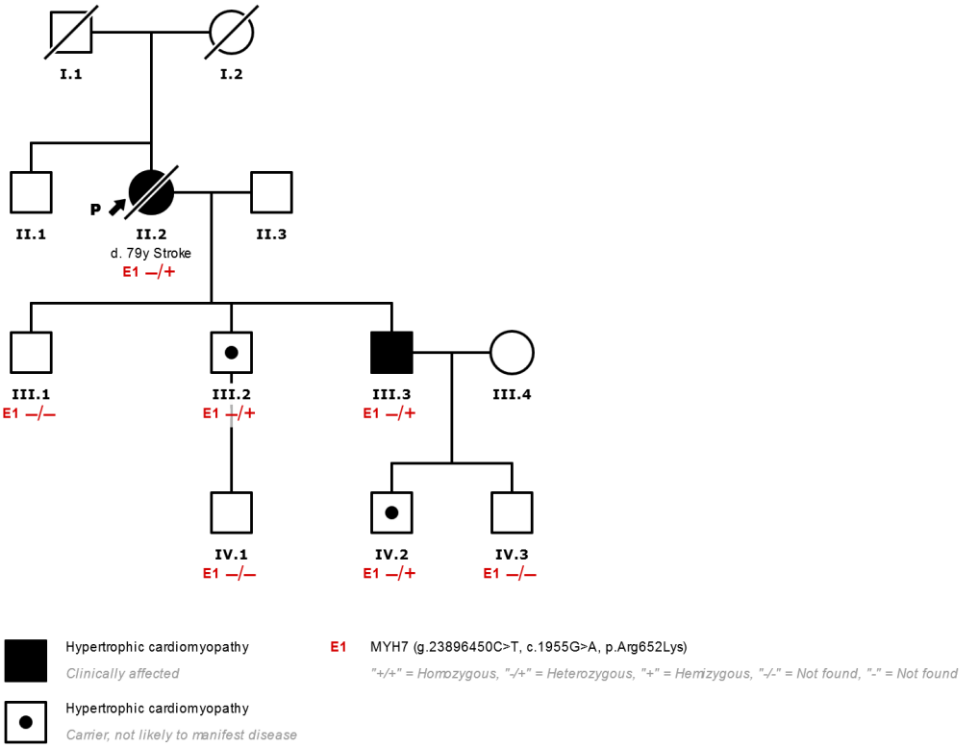

Figure 2. Family tree Nº 2. II.2 corresponds to index case Nº 2 in Table 1.
Full black patients correspond to HCM affected patients. Patients with the p.Arg652Lys in MYH7 variant and no HCM phenotype are marked with a central black mark. ACS: Acute coronary syndrome. - / -: Variant not found. - / +: Heterozygous variant found.

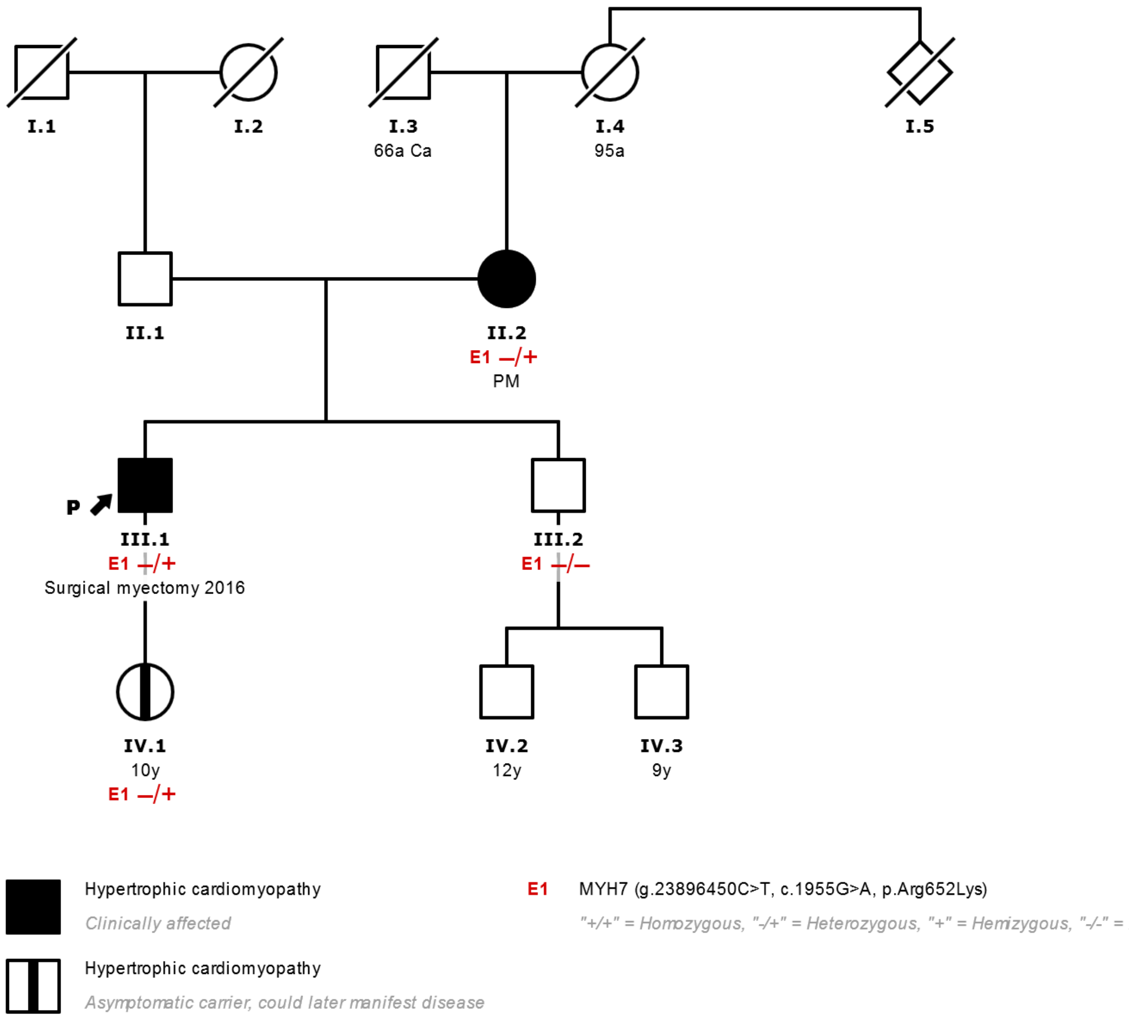

Figure 3. Family tree Nº 3. III.1 corresponds to index case Nº 3 in Table 1.
Full black patients correspond to HCM affected patients. Patients with the p.Arg652Lys in MYH7 variant and no HCM phenotype are marked with a central black mark. PM: Pacemaker. - / -: Variant not found. - / +: Heterozygous variant found.

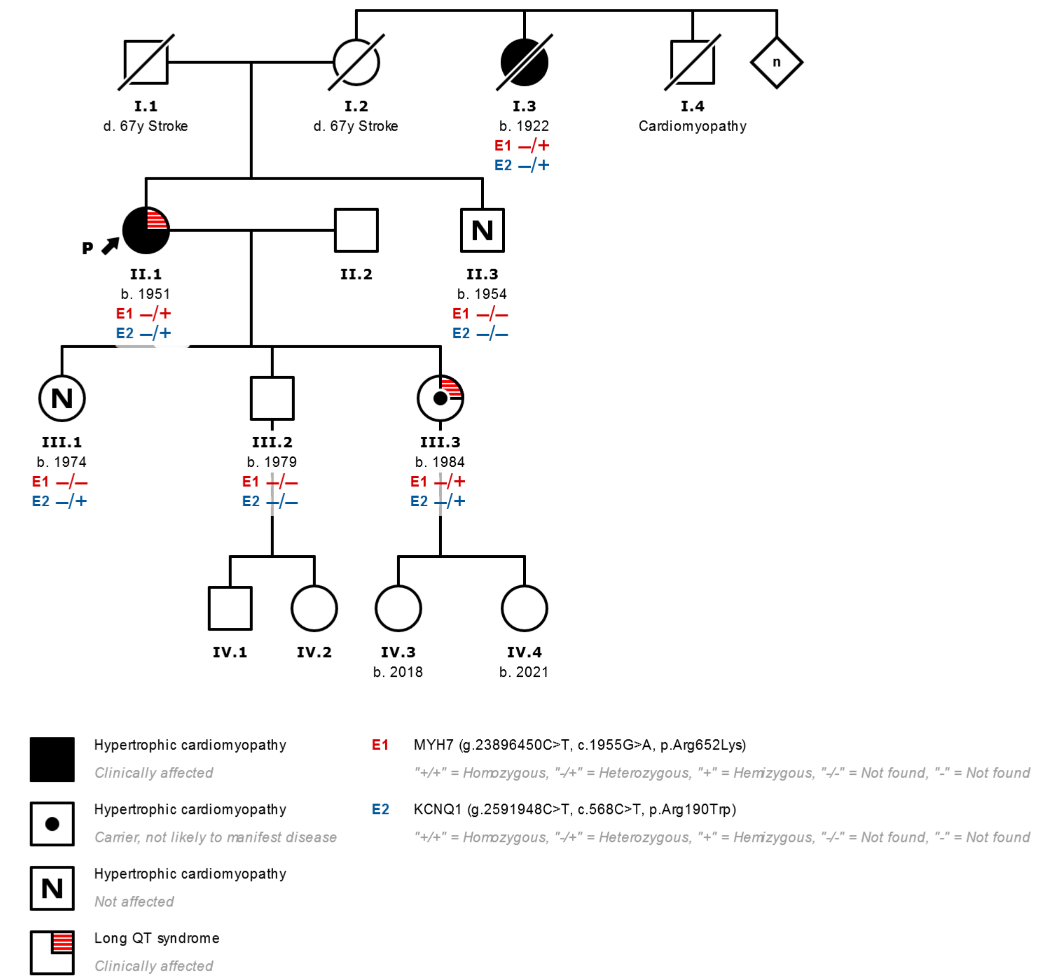

Figure 4. Family tree Nº 4. II.1 corresponds to index case Nº 4 in Table 1.
Note that two variants (E1 and E1) were found on index case and analyzed in family members afterwards. Full black patients correspond to HCM affected patients. Patients with the p.Arg652Lys in MYH7 variant and no HCM phenotype are marked with a central black mark. Red top-right corner corresponds to Long QT Syndrome affected patients. - / -: Variant not found. - / +: Heterozygous variant found.

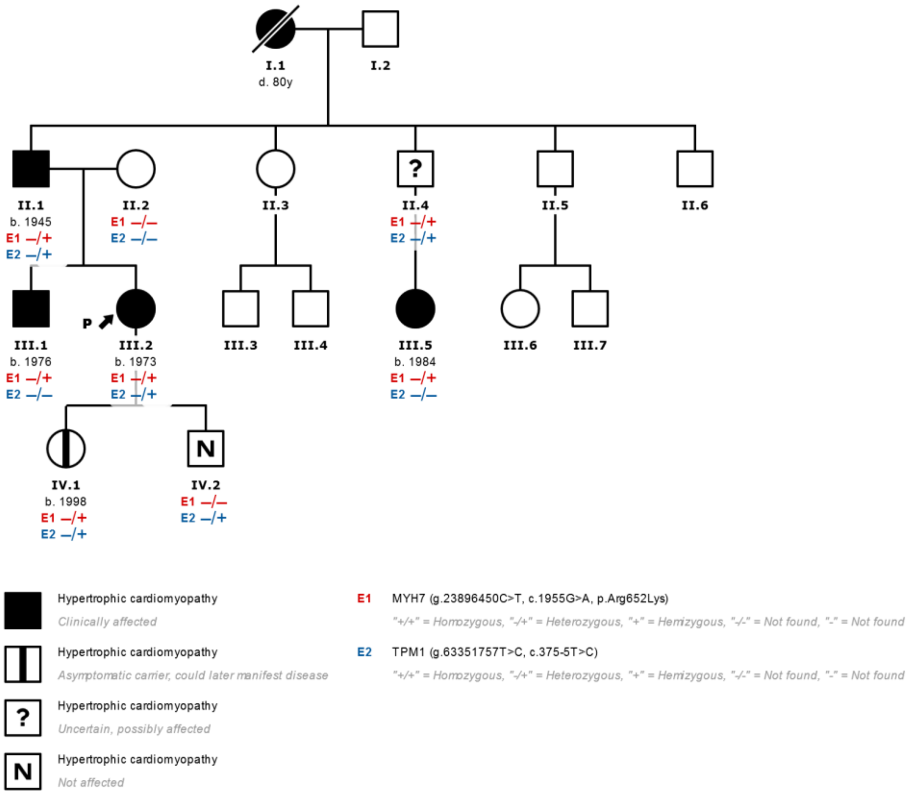

Figure 5. Family tree Nº 5. III.2 corresponds to index case Nº 5 in Table 1.
Note that two variants (E1 and E1) were found on index case and analyzed in family members afterwards. Full black patients correspond to HCM affected patients. Patients with the p.Arg652Lys in MYH7 variant and no HCM phenotype are marked with a central black mark. ACS: Acute coronary syndrome. - / -: Variant not found. - / +: Heterozygous variant found.

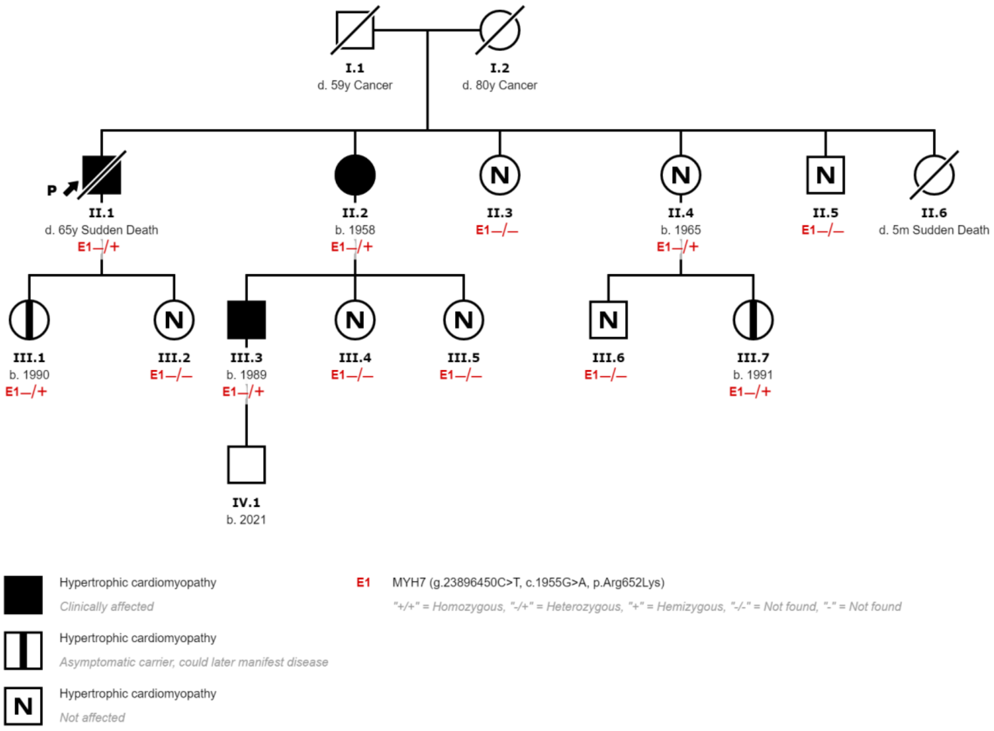

Figure 6. Family tree Nº 6. II.1 corresponds to index case Nº 6 in Table 1.
Full black patients correspond to HCM affected patients. Patients with the p.Arg652Lys in MYH7 variant and no HCM phenotype are marked with a central black mark.

- / -: Variant not found. - / +: Heterozygous variant found.

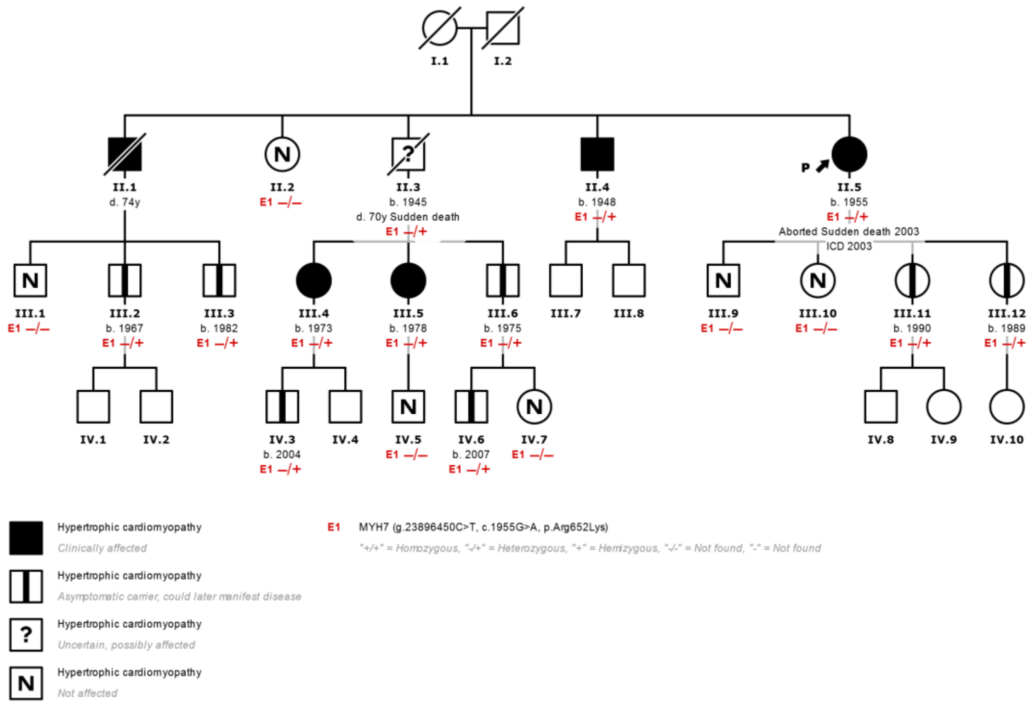

Figure 7. Family tree Nº 7. II.5 corresponds to index case Nº 7 in Table 1.
Full black patients correspond to HCM affected patients. Patients with the p.Arg652Lys in MYH7 variant and no HCM phenotype are marked with a central black mark. ACS: Acute coronary syndrome. ICD: Implantable Cardiac Defibrillator. - / -: Variant not found. - / +: Heterozygous variant found.

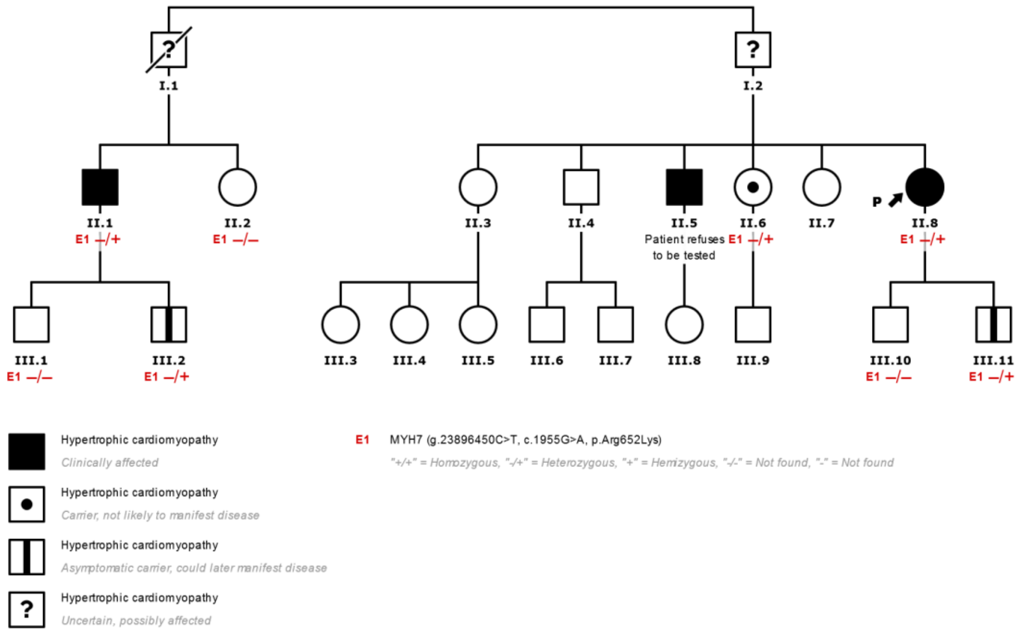

Figure 8. Family tree Nº 8. II.8 corresponds to index case Nº 8 in Table 1.
Full black patients correspond to HCM affected patients. Patients with the p.Arg652Lys in MYH7 variant and no HCM phenotype are marked with a central black mark. - / -: Variant not found. - / +: Heterozygous variant found.
